# Establishing comprehensive anesthesia services for cancer surgery at SKMCH Peshawar: Challenges, achievements, and innovations

**DOI:** 10.12669/pjms.41.4.11139

**Published:** 2025-04

**Authors:** Faraz Mansoor, Salma Jan, Zain Ul Abidin, Hafiz Rohan Ahmed Khan

**Affiliations:** 1 Faraz Mansoor, FCPS (Anesthesia), FCPS (Critical Care Medicine), Department of Anesthesia, Shaukat Khanum Memorial Cancer Hospital and Research Center, Hayatabad Phase 5, Peshawar, Pakistan; 2 Salma Jan, FCPS (Anesthesia), Department of Anesthesia, Shaukat Khanum Memorial Cancer Hospital and Research Center, Hayatabad Phase 5, Peshawar, Pakistan; 3 Zain UI Abidin, FCPS (Anesthesia), Department of Anesthesia, Shaukat Khanum Memorial Cancer Hospital and Research Center, Hayatabad Phase 5, Peshawar, Pakistan; 4 Hafiz Rohan Ahmed Khan, (Chief Resident), Department of Anesthesia, Shaukat Khanum Memorial Cancer Hospital and Research Center, Hayatabad Phase 5, Peshawar, Pakistan

**Keywords:** Cancer surgery, Developing country, New anesthesia services

## Abstract

This article details the development of new anesthesia services at Shaukat Khanum Memorial Cancer Hospital and Research Center in Peshawar, Pakistan. This involved recruiting skilled staff, who were supported by intensive training. Pre-anesthesia evaluation clinics were established to ensure patient safety and reduce surgical cancelations. Continuous professional development and innovative pain management strategies were introduced to enhance service quality, positioning SKMCH & RC as a leader in cancer-related anesthesia services in the region.

## BACKGROUND

It is projected that more than 80% of newly diagnosed cancer patients will undergo some form of cancer-related surgery.^1^ In alignment with the strategic expansion of the Shaukat Khanum Memorial Cancer Hospital and Research Center in Peshawar, Pakistan, the executive leadership of the Trust resolved to incorporate cancer surgery and non-operating room anesthesia services into Peshawar facility. Consequently, an Anesthesia team was established. Over the preceding four years, this endeavor has represented a dynamic journey characterized by the encounter of diverse challenges and the attainment of predetermined objectives.

### Recruitment and Training:

This involved the recruitment of skilled anesthesiologists and anesthesia technicians, complimented by intensive training. Both the consultants had more than five years of experience at SKMCH Lahore. Prior to their deployment to Peshawar, newly recruited anesthesia technicians underwent a comprehensive one-year training program at our Lahore center. Special attention was directed towards ensuring compliance with the rigorous standards stipulated by the Joint Commission International. After two years, in response to the escalating workload, the workforce was augmented by the recruitment of two additional anesthesia consultants. Subsequent assessment of staff retention rates after a three-year interval revealed a commendable figure exceeding 90%.

### Hospital orientation and ACLS training program:

Regular orientation sessions cover diverse topics such as patient safety, professional behavior, and effective healthcare delivery. The Quality and Patient Safety Department training sessions stresses maintaining high standards of care and safety, including discussions on radiation protection, infection control, addressing sexual harassment and appropriate conduct fosters a respectful working environment. Education on patient rights empowers both patients and staff. The hospital mandates biennial recertification in Advanced Cardiovascular Life Support (ACLS) for its personnel. These orientation and teaching sessions are crucial for equipping newly recruited staff with the professional skills and knowledge.^2^

### Pre-anesthesia clinics:

Pre-anesthesia evaluation clinics in the outpatient department coupled with direct referral mechanisms for those patients who were visiting for their surgical evaluation and coming from remote locations, has yielded profound results in ensuring patient safety and efficacy of anesthesia services. Two anesthesia clinics were established on different days within the outpatient department on weekly basis. Consultant-led patient encounters during these clinics facilitate comprehensive assessment of comorbidities and more importantly the identification of high-risk individuals, leading to informed decision making, and preoperative optimization. A standardized anesthesia assessment template is available in the Hospital Information System which serves to ensure that all patients undergo evaluation according to predefined standards.^3^ This proactive approach has resulted in negligible cancellation rates and minimal unexpected mortality.^4^

### Non-operating Room Anesthesia Services:

The Non-Operating Room Anesthesia patients has demonstrated an upward trend in the recent years due to the non-operability issues.^5^ Patients with underlying airway tumors and those with a history of radiation therapy in the head and neck region may present challenges and such cases should be labeled as high-risk. CT and MRI scans of head and neck, and thorax are helpful in airway evaluation. Each case should be carefully evaluated and anesthesia plan documented in the notes. Written consent should be signed before the start of the procedure, risks and benefits clearly explained to the patients or their parents/guardians. After the procedure these patients are shifted to the Post-anesthesia Care unit for recovery where they are discharged with written and verbal instructions once they meet the discharge criteria. Clinical audits of these services should be performed on annual basis. A modified WHO patient safety checklist is mandatory for NORA cases.

### Quality Improvement:

There is no doubt that the clinical audits play a very crucial role in maintaining high standards of healthcare delivery. Within anesthesia services, key performance indicators are meticulously monitored to ensure patient comfort and smooth recovery from anesthesia. Additionally, adherence to rigorous standards is affirmed through accreditation by the organizations like the Joint Commission International (JCI). The initial accreditation of SKMCH & RC, Peshawar in 2019 was followed by a significant accomplishment as it successfully underwent reaccreditation in 2022.

### Anesthesia equipment:

This included three anesthesia machines, notably featuring an MRI compatible unit, a dedicated difficult airway trolley which was complemented by drug trolleys in both operation rooms, ensuring stringent medication safety standards. Additionally, two video laryngoscopes enhanced procedural capabilities, while each theater boasted backup oxygen cylinders with type C circuits and portable cardiac monitors for patient transport. Two flexible fibreoptic bronchoscopes were integral to airway management, alongside thoracic epidural sets, nerve block needles, infusion pumps, and an ultrasound machine, underscoring our commitment to comprehensive perioperative care. Further, we ensured availability of various endotracheal tubes, including double lumen tubes for single lung ventilation, to cater to diverse patient needs and procedural requirements. It is the policy of the hospital that all the necessary anesthesia related equipment is regularly evaluated and serviced as required per the manufacturer instructions. Special attention is paid to the decontamination, disinfection and sterilization of the anaesthesia equipment. This practice is in accordance with the current anaesthetic equipment safety guidelines.^6^ Patient warming blankets, mattresses and fluid warming devices are available to prevent perioperative hypothermia. One crash cart is dedicated to the operation theaters.

### Pre-operative holding area and post-anesthesia care unit:

Pre-operative holding area is designed to provide a calming and friendly environment. Incorporating play spaces significantly reduces stress and promotes a sense of normalcy in pediatric population. An informed consent is taken, baseline vital signs are recorded and more importantly patient identification is confirmed. Presently, the holding bay area consists of four beds for two functional operating rooms. A fully equipped and staffed, four bedded post-anesthesia care unit provides services to the patients after surgery. Modified Aldrete score criteria are used to assess their readiness for discharge from the PACU.^7^

### Surgical population:

Anesthesia services are offered to a wide range of surgical specialties, majority cancer but also a small non-cancer patient. For thoracic surgery, incentive spirometery is introduced in the preoperative phase. This is particularly helpful in preventing postoperative respiratory complications in thoracic surgery. Thoracic epidural is preferred in these cases for postoperative pain relief.^11^ Central venous access is not recommended to measure central venous pressure in patients undergoing liver resection. Care should be taken in head and neck surgery patients and those with a history of radiation to the head and neck areas as mouth opening may be inadequate. Long durations of Trendelenburg position is usually safe, provided pressure areas are well padded and experienced surgical technicians are present in the OR. Daily ward pain round is performed by the consultant, junior anesthesia doctors and pain nurse to ensure that postoperative pain control is optimal. High risk surgical patients are postoperatively transferred to the high dependency unit to monitor and prevent against complications.

### Continuous professional development:

All consultants are required to attend one international conference on annual basis and regularly publish their research work in indexed medical journals of good repute. Shaukat Khanum Annual Cancer Symposium is considered one of the largest cancer diseases related event in this part of the world which provides a platform for healthy discussions and share clinical experience. Junior doctors who lack previous anesthesia experience undergo a structured airway training program.

## CONCLUSION

The successful establishment of anesthesia services at SKMCH Peshawar highlights the importance of strategic planning, specialized training, and adherence to international standards. This initiative has enhanced patient safety, improved surgical outcomes, and set a benchmark for cancer-related anesthesia care in the region, demonstrating a suitable model for future growth.

### Authors` Contribution:

**FM:** conceived, designed and did manuscript writing and is responsible for the integrity of the work.

**SJ and ZA:** Critical review.

**HRAK:** did reference management.
